# Calculation Formulas and Simulation Algorithms for Entropy of Function of LR Fuzzy Intervals

**DOI:** 10.3390/e21030289

**Published:** 2019-03-18

**Authors:** Jie Shen, Jian Zhou

**Affiliations:** School of Management, Shanghai University, Shanghai 200444, China

**Keywords:** entropy, LR fuzzy intervals, monotone function, calculation formulas, fuzzy simulations

## Abstract

Entropy has continuously arisen as one of the pivotal issues in optimization, mainly in portfolios, as an indicator of risk measurement. Aiming to simplify operations and extending applications of entropy in the field of LR fuzzy interval theory, this paper first proposes calculation formulas for the entropy of function via the inverse credibility distribution to directly calculate the entropy of linear function or simple nonlinear function of LR fuzzy intervals. Subsequently, to deal with the entropy of complicated nonlinear function, two novel simulation algorithms are separately designed by combining the uniform discretization process and the numerical integration process with the proposed calculation formulas. Compared to the existing simulation algorithms, the numerical results show that the advantage of the algorithms is well displayed in terms of stability, accuracy, and speed. On the whole, the simplified calculation formulas and the effective simulation algorithms proposed in this paper provide a powerful tool for the LR fuzzy interval theory, especially in entropy optimization.

## 1. Introduction

Entropy, which made its debut in 1948, was designed by Shannon [1] to measure the randomness or uncertainty of a random phenomenon. Since then, entropy has been extensively applied to various fields such as physics, finance, medicine, artificial intelligence, and so on. Philippatos and Wilson [2] scored a first by using entropy in portfolio selection problems and illustrated the construction of mean-entropy efficient portfolios. Simonelli [3] considered the entropy as an increased portfolio risk and verified the effectiveness of entropy. In addition, Xiao and Qin [4] employed a weighted combination method based on the belief entropy function [5] to solve the evidence conflict problem in multi-sensor data fusion. Guan et al. [6] presented a maximum entropy solution for implementing active queue management. In addition, Gustafsson et al. [7] used maximum entropy to improve the Bayesian credibility interval for classifier error rates. In the field of optimization, Zhang et al. [8] summarized entropy used in crowd analysis and concluded that it can effectively deal with the issues related to crowd abnormal behavior detection, crowd counting and density estimation, and crowd motion segmentation. Khan et al. [9] emphasized the optimal estimations of auxiliary variables including the entropy generation modeled via second thermodynamics law to solve flow problems. Besides, Wang et al. [10] presented a Renyi mean-entropy-skewness information criterion so that the scheduling optimization problems in flexible manufacturing systems can be handled.

It should be noted that studies above are all conducted under the probabilistic framework, which needs to collect large amounts of sample data to figure out probability distributions. However, there are many non-random factors in reality such as vagueness and ambiguity, associated in a natural way with different types of linguistic expressions, which makes the research of entropy under the probabilistic framework not yet suitable. As another study on entropy under uncertain environments, Zadeh [11] proposed new operations for the calculus of logic and initially mentioned entropy in his theory of fuzzy sets in 1965. Zadeh [12] further defined the entropy as a weighted Shannon entropy to quantify the fuzziness of an event. Moreover, Criadoa and Gachechiladzeb [13] considered the uncertainty of possibility to describe Zadeh’s general formula for the entropy. Meanwhile, there exist many applications of entropy under the possibility theory. For instance, when distributions were multimodal or discontinuous, Smaldino [14] adopted entropy to characterize the uncertainty regarding the state of the environment, which was faced by moving organisms. Liu and Zhang [15] proposed a general multi-period fuzzy portfolio optimization model with return demand which involved the entropy based on possibility measure to maximize both terminal and cumulative diversification. Additionally, Yin et al. [16] provided a possibility-based robust design optimization framework in which the entropy of the fuzzy system response was regarded as the variability index for the uncertain structural-acoustic system.

The possibility measure has properties including normality, nonnegativity and maximality [17], bar self-duality. Since self-duality is of utmost importance in both theory and practice, adhering to human’s intuition to a large extent, Liu and Liu [18] creatively defined the credibility measure, which equals the average value of the possibility measure and the necessity measure. In accordance with the credibility measure, Li and Liu [19] introduced the entropy to depict the uncertainty associated with fuzzy variables. Since then, a handful of theoretical research on entropy under the credibility theory is carried out (see, e.g., [20,21]). Recently, Rahimi et al. [22] provided novel definitions of entropy of intuitionistic fuzzy variables based on the notion of credibility under intuitionistic fuzzy environment. According to credibility measure, Zhou et al. [23] studied the entropy of LR fuzzy numbers, and the calculation formulas together with related concepts were proposed in great detail.

Meanwhile, the entropy derived from credibility measure was also employed in decision-making process. Table 1 displays the application literature about credibility theory, summarizing the objects processed by the entropy of function $H[f\left(\xi_{1},\xi_{2},\cdots ,\xi_{n}\right)]$, including the types of fuzzy variables $\xi_{i}$ and function *f*, and further considers whether the entropy of function can be clearly and analytically expressed excluding the measure operator. Most of the current literature tackled the entropy of linear function of fuzzy variables with the same type in portfolios, and thus the explicit expression of entropy of function could be obtained with no difficulty from the arithmetic operation linearity of fuzzy variables. Differently, Li [21] studied the entropy of linear function containing different types of fuzzy variables. Since it was difficult to obtain the explicit expression of entropy in this case, fuzzy simulation was used by Li [21] to solve this problem.

Fuzzy simulation is a widely accepted technique for simulating approximations, which has been studied by plenty of scholars since it was introduced by Bruckley and Hayashi [30] to solve fuzzy optimization problems in 1994. Liu and Iwamura [31] designed the fuzzy simulation whose basic idea was to convert continuous fuzzy numbers into discrete fuzzy ones by stochastically generating sample points to deal with the fuzzy chance constrained programming. After that, Liu [32] introduced this technique to solve different fuzzy programming problems based on the concept of credibility, relying on randomly generating discrete membership degrees. Huang [33] then took the lead in fuzzy simulation for entropy (FSE) based on Liu [32]’s idea, and innovatively performed stochastic discretization simulation on the entropy-integrated function, that is, the credibility function. Later, Li [21] also simulated the entropy of function to handle the entropy optimization models involving entropy of general function through stochastic discretization process in [32], which assumed that the joint credibility function was known (the simulation algorithm is denoted as FSE* in this paper). Table 2 shows the function studied in [21] and [33], as well as the principles and accuracy of the algorithms. Since the stochastic discretization simulation in Liu [32] would inevitably lead to inaccurate and lower membership degrees especially when the number of fuzzy variables involved in the function increases as pointed out in Miao et al. [34], the two existing simulation algorithms FSE and FSE* are both with low accuracy.

According to the literature mentioned above, there is a paucity of theoretical research of entropy based on credibility measure. Also, the application research is basically aimed at linear functions, merely involving simple triangular or trapezoidal fuzzy numbers of the same category, thus it is mainly applied in portfolios. Moreover, there is no research on entropy of nonlinear function. Attracted by this limited status, and considering that LR fuzzy intervals summarize triangle, trapezium, Gauss, and other nonlinear curves in fuzzy set theory, this paper is aimed at improving the calculation of fuzzy entropy of functions with respect to LR fuzzy intervals by simplifying the operation formulas and designing novel simulation algorithms from an entirely different perspective so as to extend the application situations of fuzzy entropy in decision-making problems. There are two main contributions in this paper. In terms of the definition for entropy pioneered by Li and Liu [19], some calculation formulas with analytical expressions for the entropy of fuzzy variable and function are derived separately by using the inverse credibility distribution (ICD), which can be used to figure out the entropy $H[f\left(\xi_{1},\xi_{2},\cdots ,\xi_{n}\right)]$ without difficulty when *f* is a linear function or a simple nonlinear function. With regard to the situation when *f* is a complicated nonlinear function, two simulation algorithms are designed to approximate the entropy of function on the basis of the presented calculation formulas, including the uniform discretization algorithm (UDA) referring to the method of generating the exact membership degree of each function value proposed by Miao et al. [34] and the numerical integration algorithm (NIA) based on the operational law of ICD proposed by Zhou et al. [35].

After the brief introduction as Section 1, this paper reviews fundamental knowledge of LR fuzzy intervals and credibility theory in Section 2. Section 3 proposes the calculation formulas for entropy of variable and function separately, and derives the linearity property of entropy operator. Section 4 describes the existing simulation algorithms in [21,33], and further provides two new algorithms to simulate the entropy of function. In Section 5, some numerical experiments are conducted to depict the performance of these algorithms from different perspectives, followed by Section 6, which summarizes conclusions.

## 2. Preliminaries

In this section, we present some preliminary concepts, theorems, and examples related to LR fuzzy intervals and monotone functions in the credibility theory, which will be employed throughout the whole paper.

**Definition** **1.** *(Dubois and Prade [36]) L (or R) is a decreasing function from $R^{+}\to \left[0,1\right]$, known as a shape function such that*

(1) L(0)=1;
(2) L(x)<1,∀x>0;
(3) L(x)>0,∀x<1;
(4) L(1)=0[orL(x)>0,∀xandL(+∞)=0].

**Definition** **2.** *(Dubois and Prade [36]) A fuzzy interval ξ is of LR-type if there exist shape functions L (for left) and R (for right), and scalers β>0,δ>0 with membership function (MF)*

(1) $$\mu \left(x\right)=\left\{\begin{array}{cc} L\left(\frac{\underline{a}-x}{\beta }\right), & \mathrm{if}\;\;x<\underline{a}\\ 1, & \mathrm{if}\;\;\underline{a}\leq x\leq \bar{a}\\ R\left(\frac{x-\bar{a}}{\delta }\right), & \mathrm{if}\;\;x>\bar{a}, \end{array}\right.$$

*which is denoted as $\xi ∼{\left(\underline{a},\bar{a},\beta ,\delta \right)}_{LR}$, where $\left[\underline{a},\bar{a}\right]$ is the core of ξ, $\underline{a}$ and $\bar{a}$ are respectively called the lower and upper modal values, β and δ are respectively called the left-hand and right-hand spreads.*

**Remark** **1.** *When $\underline{a}=\bar{a}$, the LR fuzzy interval degenerate to an LR fuzzy number, e.g., a triangular fuzzy number or a Gaussian fuzzy number [37]. In other words, the LR fuzzy number is a special case of LR fuzzy interval discussed in this paper.*

For an LR fuzzy interval ξ defined in Definition 2, the shape functions *L* and *R* determine its type, while the parameters $\underline{a},\bar{a},\beta ,\delta$ determine its position and spreads. Examples 1–4 provide some commonly used LR fuzzy intervals with the same parameter setting and the different shape functions, including trapezoidal fuzzy numbers, parabolic fuzzy intervals, normal fuzzy intervals, and mixture fuzzy intervals. These fuzzy intervals will be used to illustrate our work in the following sections.

**Figure 1:**
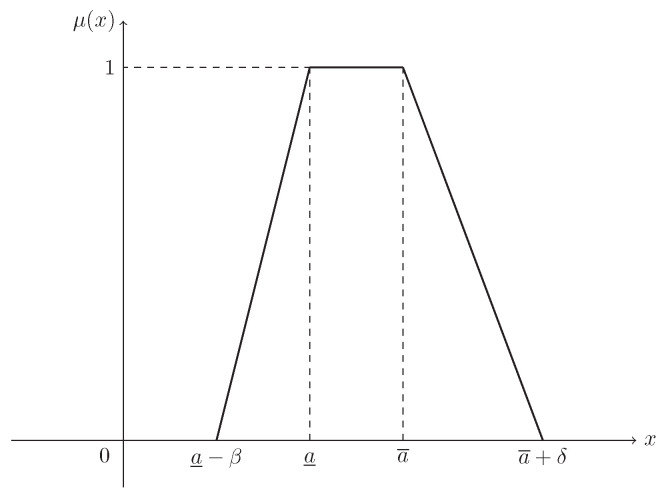
The MF in Example 1. μ(x) is the MF in Equation (2) of trapezoidal fuzzy number $\xi ∼T(\underline{a},\bar{a},\beta ,\delta )$.

**Example** **1.** *Supposing that L(x)=R(x)=max{0,1−x}, then ξ is known as a trapezoidal fuzzy number, denoted by $T(\underline{a},\bar{a},\beta ,\delta )$, and its MF is (see Figure 1)*

(2) $$\mu \left(x\right)=\left\{\begin{array}{cc} \frac{x-(\underline{a}-\beta )}{\beta }, & \mathrm{if}\;\;\underline{a}-\beta \leq x<\underline{a}\\ 1, & \mathrm{if}\;\;\underline{a}\leq x\leq \bar{a}\\ \frac{(\bar{a}+\delta )-x}{\delta }, & \mathrm{if}\;\;\bar{a}<x\leq \bar{a}+\delta . \end{array}\right.$$

**Figure 2:**
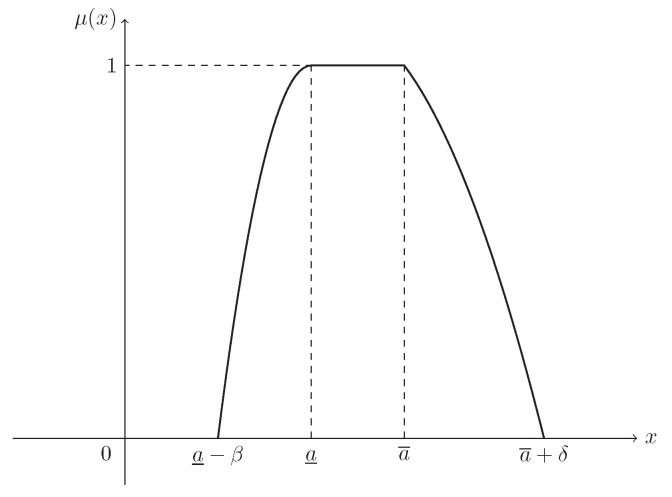
The MF in Example 2. μ(x) is the MF in Equation (3) of parabolic fuzzy interval $\xi ∼P(\underline{a},\bar{a},\beta ,\delta )$.

**Example** **2.** *If $L\left(x\right)=R\left(x\right)=\max\left\{0,1-x^{2}\right\}$, then ξ is known as a parabolic fuzzy interval, denoted by $P(\underline{a},\bar{a},\beta ,\delta )$, and its MF is (see Figure 2)*

(3) $$\mu \left(x\right)=\left\{\begin{array}{cc} 1-{\left(\frac{\underline{a}-x}{\beta }\right)}^{2}, & \mathrm{if}\;\;\underline{a}-\beta \leq x<\underline{a}\\ 1, & \mathrm{if}\;\;\underline{a}\leq x\leq \bar{a}\\ 1-{\left(\frac{x-\bar{a}}{\delta }\right)}^{2}, & \mathrm{if}\;\;\bar{a}<x\leq \bar{a}+\delta . \end{array}\right.$$

**Figure 3:**
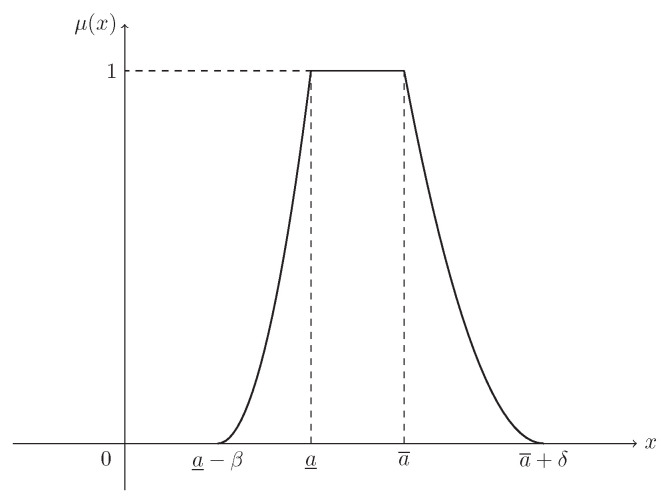
The MF in Example 3. μ(x) is the MF in Equation (4) of normal fuzzy interval $\xi ∼N(\underline{a},\bar{a},\beta ,\delta )$.

**Example** **3.** *Suppose that $L\left(x\right)=R\left(x\right)=\max\{0,{\left(1-x\right)}^{2}\}$. Then ξ is known as a normal fuzzy interval, denoted by $N(\underline{a},\bar{a},\beta ,\delta )$, with (see Figure 3)*

(4) $$\mu \left(x\right)=\left\{\begin{array}{cc} {\left(\frac{x-(\underline{a}-\beta )}{\beta }\right)}^{2}, & \mathrm{if}\;\;\underline{a}-\beta \leq x<\underline{a}\\ 1, & \mathrm{if}\;\;\underline{a}\leq x\leq \bar{a}\\ {\left(\frac{(\bar{a}+\delta )-x}{\delta }\right)}^{2}, & \mathrm{if}\;\;\bar{a}<x\leq \bar{a}+\delta . \end{array}\right.$$

**Figure 4:**
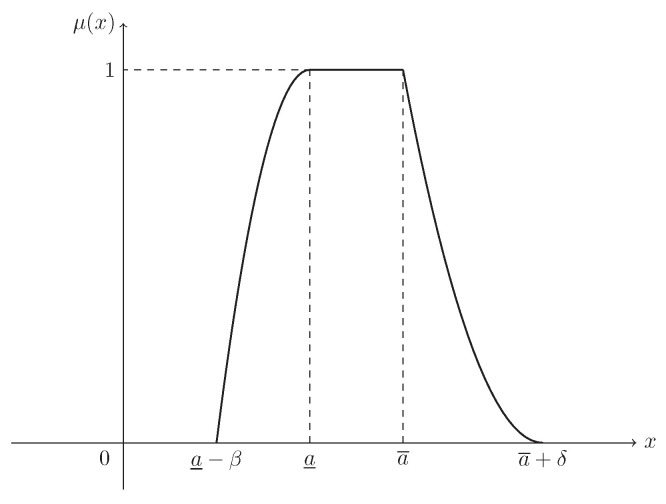
The MF in Example 4. μ(x) is the MF in Equation (5) of mixture fuzzy interval $\xi ∼M(\underline{a},\bar{a},\beta ,\delta )$.

**Example** **4.** *Suppose that $L\left(x\right)=\max\left\{0,1-x^{2}\right\}$ and $R\left(x\right)=\max\{0,{\left(1-x\right)}^{2}\}$. Then ξ is known as a mixture fuzzy interval, denoted by $M(\underline{a},\bar{a},\beta ,\delta )$, and its MF is (see Figure 4)*

(5) $$\mu \left(x\right)=\left\{\begin{array}{cc} 1-{\left(\frac{\underline{a}-x}{\beta }\right)}^{2}, & \mathrm{if}\;\;\underline{a}-\beta \leq x<\underline{a}\\ 1, & \mathrm{if}\;\;\underline{a}\leq x\leq \bar{a}\\ {\left(\frac{(\bar{a}+\delta )-x}{\delta }\right)}^{2}, & \mathrm{if}\;\;\bar{a}<x\leq \bar{a}+\delta . \end{array}\right.$$

**Definition** **3.** *(Liu and Liu [18]) Assuming that ξ is a fuzzy variable with MF, μ, and B is a set, the credibility of fuzzy event {ξ∈B} is defined by*

(6) $$\mathrm{Cr}\left\{\xi \in B\right\}=\frac{1}{2}\left(\underset{x\in B}{\sup}\mu \left(x\right)+1-\underset{x\notin B}{\sup}\mu \left(x\right)\right).$$

**Definition** **4.** *(Liu [38]) The credibility distribution (CD) Φ:[−∞,+∞]→[0,1] of a fuzzy variable ξ is defined as*

(7) Φ(x)=Cr{ξ≤x}.

As for an LR fuzzy interval $\xi ∼{\left(\underline{a},\bar{a},\beta ,\delta \right)}_{LR}$ with MF, μ, in Equation (1), on account of Equations (6) and (7), its CD can be derived as

(8) $$\Phi \left(x\right)=\left\{\begin{array}{cc} \frac{1}{2}L\left(\frac{\underline{a}-x}{\beta }\right), & \mathrm{if}\;x<\underline{a}\\ 0.5, & \mathrm{if}\;\underline{a}\leq x\leq \bar{a}\\ 1-\frac{1}{2}R\left(\frac{x-\bar{a}}{\delta }\right), & \mathrm{if}\;x>\bar{a}. \end{array}\right.$$

In view of the continuity and monotonicity of CDs of LR fuzzy intervals, Zhou et al. [35] defined a special type of LR fuzzy interval, which is called the regular LR fuzzy interval.

**Definition** **5.** *(Zhou et al. [35]) An LR fuzzy interval ξ is said to be regular if its CD, Φ(x), is continuous on {x|0<Φ(x)<1} and strictly increasing on {x|0<Φ(x)<0.5or0.5<Φ(x)<1}.*

**Definition** **6.** *(Zhou et al. [35]) Suppose that $\xi ∼{\left(\underline{a},\bar{a},\beta ,\delta \right)}_{LR}$ is a regular LR fuzzy interval. Then its inverse credibility distribution (ICD) is defined as*

(9) $$\Phi^{-1}\left(\alpha \right)=\left\{\begin{array}{cc} \underline{a}-\beta L^{-1}\left(2\alpha \right), & \mathrm{if}\;\;0<\alpha <0.5\\ {}[\underline{a},\bar{a}], & \mathrm{if}\;\;\alpha =0.5\\ \bar{a}+\delta R^{-1}\left(2-2\alpha \right), & \mathrm{if}\;\;0.5<\alpha <1. \end{array}\right.$$

Please note that the ICD, $\Phi^{-1}\left(\alpha \right)$, is well defined on the domain (0,1). If required, the open interval can be extended by

$$\Phi^{-1}\left(0\right)=\lim_{\alpha ↓0}\Phi^{-1}\left(\alpha \right),\;\Phi^{-1}\left(1\right)=\lim_{\alpha ↑1}\Phi^{-1}\left(\alpha \right).$$

Based on Definition 5, it is clear that the four kinds of LR fuzzy intervals given in Examples 1–4 are all regular LR fuzzy intervals. Thus, in the light of Equations (8) and (9), Examples 5–8 derive their CDs and ICDs, respectively, for our purpose.

**Figure 5:**
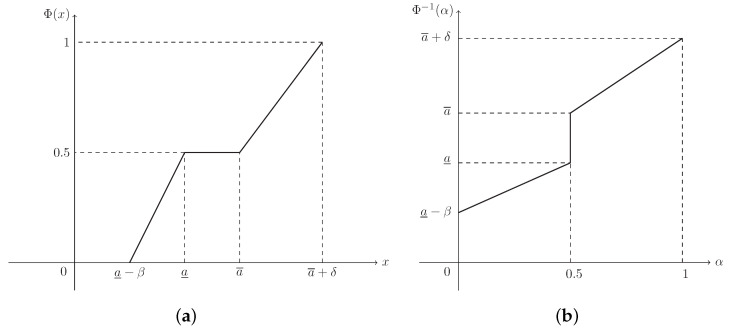
The CD and ICD of trapezoidal fuzzy number $\xi ∼T(\underline{a},\bar{a},\beta ,\delta )$ in Example 5. (**a**) Φ(x) is the CD in Equation (10); (**b**) $\Phi^{-1}\left(\alpha \right)$ is the ICD in Equation (11).

**Example** **5.** *The CD of trapezoidal fuzzy number $\xi ∼T(\underline{a},\bar{a},\beta ,\delta )$ in Example 1 is (see Figure 5a)*

(10) $$\Phi \left(x\right)=\left\{\begin{array}{cc} \frac{x-(\underline{a}-\beta )}{2\beta }, & \mathrm{if}\;\;\underline{a}-\beta \leq x<\underline{a}\\ 0.5, & \mathrm{if}\;\;\underline{a}\leq x\leq \bar{a}\\ \frac{x-(\bar{a}-\delta )}{2\delta }, & \mathrm{if}\;\;\bar{a}<x\leq \bar{a}+\delta , \end{array}\right.$$

*and its ICD is (see Figure 5b)*

(11) $$\Phi^{-1}\left(\alpha \right)=\left\{\begin{array}{cc} 2\beta \alpha +\underline{a}-\beta , & \mathrm{if}\;\;0\leq \alpha <0.5\\ {}[\underline{a},\bar{a}], & \mathrm{if}\;\;\alpha =0.5\\ 2\delta \alpha +\bar{a}-\delta , & \mathrm{if}\;\;0.5<\alpha \leq 1. \end{array}\right.$$

**Figure 6:**
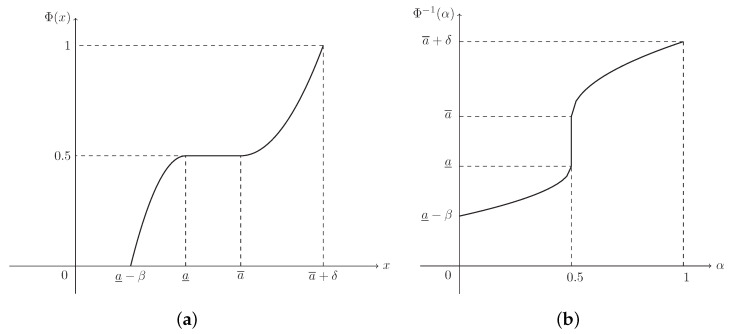
The CD and ICD of parabolic fuzzy interval $\xi ∼P(\underline{a},\bar{a},\beta ,\delta )$ in Example 6. (**a**) Φ(x) is the CD in Equation (12); (**b**) $\Phi^{-1}\left(\alpha \right)$ is the ICD in Equation (13).

**Example** **6.** *The CD of parabolic fuzzy interval $\xi ∼P(\underline{a},\bar{a},\beta ,\delta )$ in Example 2 is (see Figure 6a)*

(12) $$\Phi \left(x\right)=\left\{\begin{array}{cc} \frac{1}{2}-\frac{1}{2}{\left(\frac{\underline{a}-x}{\beta }\right)}^{2}, & \mathrm{if}\;\;\underline{a}-\beta \leq x<\underline{a}\\ 0.5, & \mathrm{if}\;\;\underline{a}\leq x\leq \bar{a}\\ \frac{1}{2}+\frac{1}{2}{\left(\frac{x-\bar{a}}{\delta }\right)}^{2}, & \mathrm{if}\;\;\bar{a}<x\leq \bar{a}+\delta , \end{array}\right.$$

*and its ICD is (see Figure 6b)*

(13) $$\Phi^{-1}\left(\alpha \right)=\left\{\begin{array}{cc} \underline{a}-\beta \sqrt{1-2\alpha }, & \mathrm{if}\;\;0\leq \alpha <0.5\\ {}[\underline{a},\bar{a}], & \mathrm{if}\;\;\alpha =0.5\\ \bar{a}+\delta \sqrt{2\alpha -1}, & \mathrm{if}\;\;0.5<\alpha \leq 1. \end{array}\right.$$

**Figure 7:**
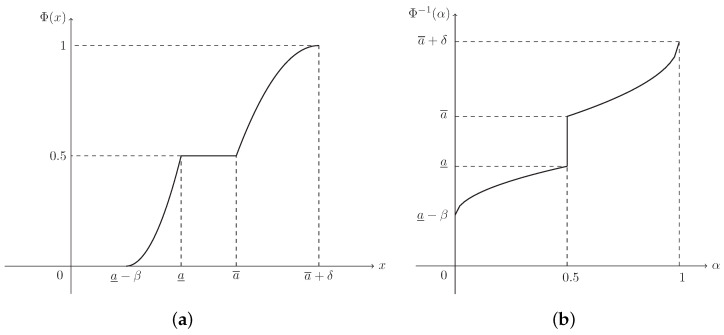
The CD and ICD of normal fuzzy interval $\xi ∼N(\underline{a},\bar{a},\beta ,\delta )$ in Example 7. (**a**) Φ(x) is the CD in Equation (14); (**b**) $\Phi^{-1}\left(\alpha \right)$ is the ICD in Equation (15).

**Example** **7.** *The CD of normal fuzzy interval $\xi ∼N(\underline{a},\bar{a},\beta ,\delta )$ in Example 3 is (see Figure 7a)*

(14) $$\Phi \left(x\right)=\left\{\begin{array}{cc} \frac{1}{2}{\left(\frac{x-(\underline{a}-\beta )}{\beta }\right)}^{2}, & \mathrm{if}\;\;\underline{a}-\beta \leq x<\underline{a}\\ 0.5, & \mathrm{if}\;\;\underline{a}\leq x\leq \bar{a}\\ 1-\frac{1}{2}{\left(\frac{(\bar{a}+\delta )-x}{\delta }\right)}^{2}, & \mathrm{if}\;\;\bar{a}<x\leq \bar{a}+\delta , \end{array}\right.$$

*and its ICD is (see Figure 7b)*

(15) $$\Phi^{-1}\left(\alpha \right)=\left\{\begin{array}{cc} \underline{a}-\beta +\beta \sqrt{2\alpha }, & \mathrm{if}\;\;0\leq \alpha <0.5\\ {}[\underline{a},\bar{a}], & \mathrm{if}\;\;\alpha =0.5\\ \bar{a}+\delta -\delta \sqrt{2-2\alpha }, & \mathrm{if}\;\;0.5<\alpha \leq 1. \end{array}\right.$$

**Figure 8:**
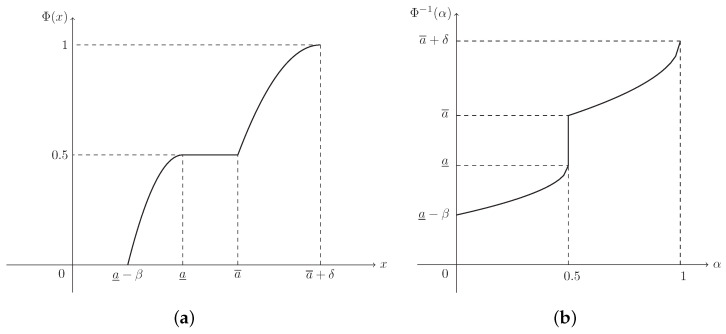
The CD and ICD of mixture fuzzy interval $\xi ∼M(\underline{a},\bar{a},\beta ,\delta )$ in Example 8. (**a**) Φ(x) is the CD in Equation (16); (**b**) $\Phi^{-1}\left(\alpha \right)$ is the ICD in Equation (17).

**Example** **8.** *The CD of mixture fuzzy interval $\xi ∼M(\underline{a},\bar{a},\beta ,\delta )$ in Example 4 is (see Figure 8a)*

(16) $$\Phi \left(x\right)=\left\{\begin{array}{cc} \frac{1}{2}-\frac{1}{2}{\left(\frac{\underline{a}-x}{\beta }\right)}^{2}, & \mathrm{if}\;\;\underline{a}-\beta \leq x<\underline{a}\\ 0.5, & \mathrm{if}\;\;\underline{a}\leq x\leq \bar{a}\\ 1-\frac{1}{2}{\left(\frac{(\bar{a}+\delta )-x}{\delta }\right)}^{2}, & \mathrm{if}\;\;\bar{a}<x\leq \bar{a}+\delta , \end{array}\right.$$

*and its ICD is (see Figure 8b)*

(17) $$\Phi^{-1}\left(\alpha \right)=\left\{\begin{array}{cc} \underline{a}-\beta \sqrt{1-2\alpha }, & \mathrm{if}\;\;0\leq \alpha <0.5\\ {}[\underline{a},\bar{a}], & \mathrm{if}\;\;\alpha =0.5\\ \bar{a}+\delta -\delta \sqrt{2-2\alpha }, & \mathrm{if}\;\;0.5<\alpha \leq 1. \end{array}\right.$$

**Definition** **7.** *(Liu [38]) A strictly monotone function $f(x_{1},x_{2},\cdots ,x_{n})$ is established when it is strictly increasing in regard to $x_{1},x_{2},\cdots ,$$x_{h}$ and strictly decreasing in regard to $x_{h+1},x_{h+2},$$\cdots ,x_{n}$, that is,*

(18) $$f\left(x_{1},\cdots ,x_{h},x_{h+1},\cdots ,x_{n}\right)\leq f\left(y_{1},\cdots ,y_{h},y_{h+1},\cdots ,y_{n}\right)$$

*whenever $x_{i}\leq y_{i}$ for i=1,2,⋯,h and $x_{i}\geq y_{i}$ for i=h+1,⋯,n, and*

(19) $$f\left(x_{1},\cdots ,x_{h},x_{h+1},\cdots ,x_{n}\right)<f\left(y_{1},\cdots ,y_{h},y_{h+1},\cdots ,y_{n}\right)$$

*whenever $x_{i}<y_{i}$ for i=1,2,⋯,h and $x_{i}>y_{i}$ for i=h+1,⋯,n.*

**Theorem** **1.** *(Zhou et al. [35]) Let $\xi_{1},\xi_{2},\cdots ,\xi_{n}$ be independent regular LR fuzzy intervals with CDs, $\Phi_{1},\Phi_{2},\cdots ,\Phi_{n}$, respectively. If the continuous function $f(x_{1},x_{2},\cdots ,x_{n})$ is strictly increasing in regard to $x_{1},\cdots ,x_{h}$ and strictly decreasing in regard to $x_{h+1},\cdots ,x_{n}$, then $\xi =f(\xi_{1},\xi_{2},\cdots ,\xi_{n})$ is a regular LR fuzzy interval with ICD,*

$$\Psi^{-1}\left(\alpha \right)=f\left(\Phi_{1}^{-1}\left(\alpha \right),\cdots ,\Phi_{h}^{-1}\left(\alpha \right),\Phi_{h+1}^{-1}\left(1-\alpha \right),\cdots ,\Phi_{n}^{-1}\left(1-\alpha \right)\right).$$

## 3. Calculation Formulas for Entropy of Monotone Function

This section first presents the definition of entropy of continuous fuzzy variable proposed by Li and Liu [19]. Then, an equivalent formula for entropy of LR fuzzy interval is derived in terms of the ICD. Ultimately, the calculation formula for entropy of monotone function of LR fuzzy intervals is presented, and the linearity of entropy operator is also verified.

### 3.1. Definition and Calculation Formula for H[ξ]

**Definition** **8.** *(Li and Liu [19]) Suppose that ξ is a continuous fuzzy variable. Then the entropy of ξ is defined as*

(20) $$\;H\left[\xi \right]=\int_{-\infty }^{+\infty }S\left(\mathrm{Cr}\{\xi =x\}\right)dx,$$

*where S(t)=−tlnt−(1−t)ln(1−t).*

According to Equations (1) and (6), the credibility function [21] of an LR fuzzy interval $\xi ∼{\left(\underline{a},\bar{a},\beta ,\delta \right)}_{LR}$ is

(21) $$\mathrm{Cr}\left\{\xi =x\right\}=\left\{\begin{array}{cc} \frac{1}{2}L\left(\frac{\underline{a}-x}{\beta }\right), & \mathrm{if}\;x<\underline{a}\\ 0.5, & \mathrm{if}\;\underline{a}\leq x\leq \bar{a}\\ \frac{1}{2}R\left(\frac{x-\bar{a}}{\delta }\right), & \mathrm{if}\;x>\bar{a}. \end{array}\right.$$

Hence, the entropy of ξ is

(22) $$H\left[\xi \right]=\int_{-\infty }^{\underline{a}}S\left(\frac{1}{2}L\left(\frac{\underline{a}-x}{\beta }\right)\right)dx+\int_{\underline{a}}^{\bar{a}}S\left(0.5\right)dx+\int_{\bar{a}}^{+\infty }S\left(\frac{1}{2}R\left(\frac{x-\bar{a}}{\delta }\right)\right)dx.$$

By using the calculation formula in Equation (22), the entropy of the four kinds of LR fuzzy intervals mentioned above is derived and presented in Examples 9–12.

**Example** **9.** *Suppose that ξ is a trapezoidal fuzzy number in Example 1. Then its entropy can be calculated as*

(23) $$\begin{array}{cc} H[\xi ] & =\int_{\underline{a}-\beta }^{\underline{a}}S\left(\frac{x-(\underline{a}-\beta )}{2\beta }\right)dx+\int_{\underline{a}}^{\bar{a}}S\left(0.5\right)dx+\int_{\bar{a}}^{\bar{a}+\delta }S\left(\frac{(\bar{a}+\delta )-x}{2\delta }\right)dx\\ & =\int_{0}^{0.5}2\beta S\left(t\right)dt+\left(\bar{a}-\underline{a}\right)\ln2-\int_{0.5}^{0}2\delta S\left(t\right)dt\\ & =\frac{\beta +\delta }{2}+\left(\bar{a}-\underline{a}\right)\ln2. \end{array}$$

*Assuming that $\underline{a}=\bar{a}$, which signifies that ξ is a triangular fuzzy number, its entropy is $H\left[\xi \right]=\frac{\beta +\delta }{2}$.*

**Example** **10.** *Suppose that ξ is a parabolic fuzzy interval in Example 2. Then its entropy can be calculated as*

(24) $$\begin{array}{cc} H[\xi ] & =\int_{\underline{a}-\beta }^{\underline{a}}S\left(\frac{1}{2}-\frac{1}{2}{\left(\frac{\underline{a}-x}{\beta }\right)}^{2}\right)dx+\int_{\underline{a}}^{\bar{a}}S\left(0.5\right)dx+\int_{\bar{a}}^{\bar{a}+\delta }S\left(\frac{1}{2}-\frac{1}{2}{\left(\frac{x-\bar{a}}{\delta }\right)}^{2}\right)dx\\ & =\int_{0}^{0.5}\beta {\left(1-2t\right)}^{-\frac{1}{2}}S\left(t\right)dt+\left(\bar{a}-\underline{a}\right)\ln2-\int_{0.5}^{0}\delta {\left(1-2t\right)}^{-\frac{1}{2}}S\left(t\right)dt\\ & =\left(\beta +\delta \right)\left(\frac{4}{3}-\frac{\pi }{6}-\frac{\ln2}{3}\right)+\left(\bar{a}-\underline{a}\right)\ln2. \end{array}$$

*Assuming that $\underline{a}=\bar{a}$, which signifies that ξ is a parabolic fuzzy number, its entropy is $H\left[\xi \right]=\left(\beta +\delta \right)\left(\frac{4}{3}-\frac{\pi }{6}-\frac{\ln2}{3}\right)$.*

**Example** **11.** *Suppose that ξ is a normal fuzzy interval in Example 3. Then its entropy can be calculated as*

(25) $$\begin{array}{cc} \;H[\xi ] & =\int_{\underline{a}-\beta }^{\underline{a}}S\left(\frac{1}{2}{\left(\frac{x-(\underline{a}-\beta )}{\beta }\right)}^{2}\right)dx+\int_{\underline{a}}^{\bar{a}}S\left(0.5\right)dx+\int_{\bar{a}}^{\bar{a}+\delta }S\left(\frac{1}{2}{\left(\frac{(\bar{a}+\delta )-x}{\delta }\right)}^{2}\right)dx\\ & =\int_{0}^{0.5}\beta {\left(2t\right)}^{-\frac{1}{2}}S\left(t\right)dt+\left(\bar{a}-\underline{a}\right)\ln2-\int_{0.5}^{0}\delta {\left(2t\right)}^{-\frac{1}{2}}S\left(t\right)dt\\ & =\left(\beta +\delta \right)\left(\frac{4}{3}-\frac{2\sqrt{2}}{3}\ln\left(3+2\sqrt{2}\right)\right)+\left(\bar{a}-\underline{a}+\beta +\delta \right)\ln2. \end{array}$$

*Assuming that $\underline{a}=\bar{a}$, which signifies that ξ is a normal fuzzy number, its entropy is $H\left[\xi \right]=\left(\beta +\delta \right)(\frac{4}{3}-\frac{2\sqrt{2}}{3}\ln\left(3+2\sqrt{2}\right)+\ln2)$.*

**Example** **12.** *Suppose that ξ is a mixture fuzzy interval in Example 4. Then its entropy can be calculated as*

(26) $$\begin{array}{cc} \;H[\xi ] & =\int_{\underline{a}-\beta }^{\underline{a}}S\left(\frac{1}{2}-\frac{1}{2}{\left(\frac{\underline{a}-x}{\beta }\right)}^{2}\right)dx+\int_{\underline{a}}^{\bar{a}}S\left(0.5\right)dx+\int_{\bar{a}}^{\bar{a}+\delta }S\left(\frac{1}{2}{\left(\frac{(\bar{a}+\delta )-x}{\delta }\right)}^{2}\right)dx\\ & =\int_{0}^{0.5}\beta {\left(1-2t\right)}^{-\frac{1}{2}}S\left(t\right)dt+\left(\bar{a}-\underline{a}\right)\ln2-\int_{0.5}^{0}\delta {\left(2t\right)}^{-\frac{1}{2}}S\left(t\right)dt\\ & =\beta \left(\frac{4}{3}-\frac{\pi }{6}-\frac{\ln2}{3}\right)+\delta \left(\frac{4}{3}-\frac{2\sqrt{2}}{3}\ln\left(2\sqrt{2}+3\right)\right)+\left(\bar{a}-\underline{a}+\delta \right)\ln2. \end{array}$$

*Assuming that $\underline{a}=\bar{a}$, which signifies that ξ is a mixture fuzzy number, its entropy is $H\left[\xi \right]=\beta \left(\frac{4}{3}-\frac{\pi }{6}-\frac{\ln2}{3}\right)+\delta \left(\frac{4}{3}+\ln2-\frac{2\sqrt{2}}{3}\ln\left(2\sqrt{2}+3\right)\right)$.*

**Theorem** **2.** *Provided that ξ is a regular LR fuzzy interval, if its entropy exists, then*

(27) $$H\left[\xi \right]=\int_{0}^{1}\Phi^{-1}\left(\alpha \right)\ln\frac{\alpha }{1-\alpha }d\alpha ,$$

*where $\Phi^{-1}$ is the ICD of ξ.* □

**Proof** **of Theorem 2.** Suppose that ξ is an LR fuzzy interval represented by $\xi ∼{\left(\underline{a},\bar{a},\beta ,\delta \right)}_{LR}$ with the CD, Φ. According to Equations (8) and (21), we can obtain

(28) $$\mathrm{Cr}\left\{\xi =x\right\}=\left\{\begin{array}{cc} \Phi (x), & \mathrm{if}\;x\leq \bar{a}\\ 1-\Phi (x), & \mathrm{if}\;x>\bar{a}. \end{array}\right.$$

In light of Definition 8 and the equation S(t)=S(1−t), the entropy can be easily calculated as

(29) $$\begin{array}{cc} \;H[\xi ] & =\int_{-\infty }^{\bar{a}}S\left(\Phi \left(x\right)\right)dx+\int_{\bar{a}}^{+\infty }S\left(1-\Phi \left(x\right)\right)dx\\ & =\int_{-\infty }^{+\infty }S\left(\Phi \left(x\right)\right)dx. \end{array}$$

Thus, we have

(30) $$\begin{array}{cc} H[\xi ] & =\int_{-\infty }^{+\infty }S\left(\Phi \left(x\right)\right)dx\\ & =\int_{-\infty }^{0}\int_{0}^{\Phi (x)}S^{\prime }\left(\alpha \right)d\alpha dx-\int_{0}^{+\infty }\int_{\Phi (x)}^{1}S^{\prime }\left(\alpha \right)d\alpha dx, \end{array}$$

where $S^{\prime }\left(\alpha \right)$ is the derivative of S(α) with $S^{\prime }\left(\alpha \right)={\left(-\alpha \ln\alpha -\left(1-\alpha \right)\ln\left(1-\alpha \right)\right)}^{\prime }=-\ln\frac{\alpha }{1-\alpha }.$ By the Fubini theorem, it immediately follows that

(31) $$\begin{array}{cc} H[\xi ] & =\int_{0}^{\Phi (0)}\int_{\Phi^{-1}\left(\alpha \right)}^{0}S^{\prime }\left(\alpha \right)dxd\alpha -\int_{\Phi (0)}^{1}\int_{0}^{\Phi^{-1}\left(\alpha \right)}S^{\prime }\left(\alpha \right)dxd\alpha \\ & =-\int_{0}^{1}\Phi^{-1}\left(\alpha \right)S^{\prime }\left(\alpha \right)d\alpha \\ & =\int_{0}^{1}\Phi^{-1}\left(\alpha \right)\ln\frac{\alpha }{1-\alpha }d\alpha . \end{array}$$

**Remark** **2.** *It should be noted that Zhou et al. [23] have verified that the calculation formulas in Equation (27) is also effective for LR fuzzy numbers. That is, the formulas derived in Theorem 2 with respect to LR fuzzy intervals is a generalized one involving Zhou et al. [23]’s result as a special case.*

By means of Theorem 2, we can calculate the entropy of the above four types of regular LR fuzzy intervals (see Examples 10–13) via their ICDs directly. More clearly, for a regular LR fuzzy interval $\xi ∼{\left(\underline{a},\bar{a},\beta ,\delta \right)}_{LR}$, its entropy can be calculated via Equations (9) and (27) as

(32) $$H\left[\xi \right]=\int_{0}^{0.5}\left(\underline{a}-\beta L^{-1}\left(2\alpha \right)\right)\ln\frac{\alpha }{1-\alpha }d\alpha +\int_{0.5}^{1}\left(\bar{a}+\delta R^{-1}\left(2-2\alpha \right)\right)\ln\frac{\alpha }{1-\alpha }d\alpha .$$

Compared with Equation (22), the calculation formula for H[ξ] in Equation (32) is much simplified.

### 3.2. Calculation Formula for $H[f\left(\xi_{1},\xi_{2},\cdots ,\xi_{n}\right)]$

According to Definition 8, it is clear that the calculation of entropy in credibility theory is complicated due to the definition itself. Hence, it is more difficult to deal with the entropy of function, that is,

(33) $$H\left[f\left(\xi_{1},\xi_{2},\cdots ,\xi_{n}\right)\right]=\int_{-\infty }^{+\infty }S\left(\mathrm{Cr}\{f\left(\xi_{1},\xi_{2},\cdots ,\xi_{n}\right)=x\}\right)dx,$$

which results in the limitations of the current research.

Referring to the existing literature about the entropy of function $H[f\left(\xi_{1},\xi_{2},\cdots ,\xi_{n}\right)]$, in terms of continuous fuzzy variable ξ, Li and Liu [19] has proved that H[aξ+b]=|a|H[ξ], where aξ+b is a linear function of the single fuzzy variable ξ. Regarding the function of fuzzy variables, $f(\xi_{1},\xi_{2},\cdots ,\xi_{n})$, where *f* is an *n*-dimensional function, almost all the current work focuses on portfolio selection problems to process the linear function of fuzzy variables. In addition, the fuzzy variables involved are either triangular fuzzy numbers or trapezoidal fuzzy numbers (see, e.g., [24,27]).

Take the trapezoidal fuzzy numbers as an example to illustrate their work. Assume that $\xi_{i}∼T\left({\underline{a}}_{i},{\bar{a}}_{i},\beta_{i},\delta_{i}\right)$, and $x_{i}\geq 0$, i=1,2,⋯,n. According to the arithmetic operation linearity of trapezoidal fuzzy numbers, it is easy to derive that

$$\xi_{1}x_{1}+\xi_{2}x_{2}+\cdots +\xi_{n}x_{n}∼T\left(\sum_{i=1}^{n}{\underline{a}}_{i}x_{i},\sum_{i=1}^{n}{\bar{a}}_{i}x_{i},\sum_{i=1}^{n}\beta_{i}x_{i},\sum_{i=1}^{n}\delta_{i}x_{i}\right).$$

Then in accordance with Equation (23), the entropy of function in portfolio can be obtained as

$$H\left[\xi_{1}x_{1}+\xi_{2}x_{2}+\cdots +\xi_{n}x_{n}\right]=\frac{1}{2}\left(\sum_{i=1}^{n}\beta_{i}x_{i}+\sum_{i=1}^{n}\delta_{i}x_{i}\right)+\left(\sum_{i=1}^{n}{\bar{a}}_{i}x_{i}-\sum_{i=1}^{n}{\underline{a}}_{i}x_{i}\right)\ln2.$$

To break the deadlock and apply fuzzy entropy to more range of areas besides portfolios, according to Theorems 1 and 2, we put forward a new calculation formula for entropy of function that can be universally applied.

**Theorem** **3.** *Let $\xi_{1},\xi_{2},\cdots ,\xi_{n}$ be independent regular LR fuzzy intervals with CDs, $\Phi_{1},\Phi_{2},\cdots ,\Phi_{n}$, respectively. If the continuous function $f(x_{1},x_{2},\cdots ,x_{n})$ is strictly increasing in regard to $x_{1},\cdots ,x_{h}$ and strictly decreasing in regard to $x_{h+1},\cdots ,x_{n}$, then the entropy of $f(\xi_{1},\xi_{2},\cdots ,\xi_{n})$ is*

(34) $$H\left[f\left(\xi_{1},\xi_{2},\cdots ,\xi_{n}\right)\right]=\int_{0}^{1}f\left(\Phi_{1}^{-1}\left(\alpha \right),\cdots ,\Phi_{h}^{-1}\left(\alpha \right),\Phi_{h+1}^{-1}\left(1-\alpha \right),\cdots ,\Phi_{n}^{-1}\left(1-\alpha \right)\right)\ln\frac{\alpha }{1-\alpha }d\alpha .$$

**Proof** **of Theorem 3.** It follows immediately from Theorems 1 and 2. □

Through this calculation formula for entropy of function, the crisp integration can be obtained by substituting the ICDs of LR fuzzy intervals (i.e., $\Phi_{i}^{-1}$, see Equation (9) in Definition 6) into Equation (34), which avoids the difficulty of calculating the entropy of function via the credibility measure Cr and the function *S* in Equation (33). In consequence, in addition to the entropy of linear function, Equation (34) can be also used to directly figure out some slightly more complex conditions, such as entropy of some simple nonlinear functions of different types of fuzzy variables.

**Example** **13.** *Let $\xi_{1}∼T\left(2,3,1,1\right)$ and $\xi_{2}∼P\left(4,5,2,2\right)$ be two independent fuzzy intervals. According to Equations (11), (13) and (34), the entropy of function $f_{1}\left(\xi_{1},\xi_{2}\right)=6\xi_{1}+2\xi_{2}$ can be calculated as*

(35) $$\begin{array}{cc} H[6\xi_{1}+2\xi_{2}] & =\int_{0}^{1}\left(6\Phi_{1}^{-1}\left(\alpha \right)+2\Phi_{2}^{-1}\left(\alpha \right)\right)\ln\frac{\alpha }{1-\alpha }d\alpha \\ & =\int_{0}^{0.5}\left(6\times \left(2\alpha +1\right)+2\times \left(4-2\sqrt{1-2\alpha }\right)\right)\ln\frac{\alpha }{1-\alpha }d\alpha \\ & \;\;+\int_{0.5}^{1}\left(6\times \left(2\alpha +2\right)+2\times \left(5+2\sqrt{2\alpha -1}\right)\right)\ln\frac{\alpha }{1-\alpha }d\alpha \\ & =\frac{50}{3}+\frac{16\ln2}{3}-\frac{4\pi }{3}, \end{array}$$

*and the entropy of function $f_{2}\left(\xi_{1},\xi_{2}\right)=6\xi_{1}\wedge 2\xi_{2}$ can be similarly obtained as*

(36) $$\begin{array}{cc} H[6\xi_{1}\wedge 2\xi_{2}] & =\int_{0}^{1}\left(6\Phi_{1}^{-1}\left(\alpha \right)\wedge 2\Phi_{2}^{-1}\left(\alpha \right)\right)\ln\frac{\alpha }{1-\alpha }d\alpha \\ & =\int_{0}^{0.5}\left(6\times \left(2\alpha +1\right)\wedge 2\times \left(4-2\sqrt{1-2\alpha }\right)\right)\ln\frac{\alpha }{1-\alpha }d\alpha \\ & \;\;+\int_{0.5}^{1}\left(6\times \left(2\alpha +2\right)\wedge 2\times \left(5+2\sqrt{2\alpha -1}\right)\right)\ln\frac{\alpha }{1-\alpha }d\alpha \\ & =\frac{32}{3}-\frac{2\ln2}{3}-\frac{4\pi }{3}. \end{array}$$

### 3.3. Linearity Property of Entropy Operator

In the following, we further verify the linearity of entropy operator with respect to regular fuzzy intervals based on Theorem 3.

**Theorem** **4.** *Let $\xi_{1},\xi_{2},\cdots ,\xi_{n}$ be independent regular LR fuzzy intervals. If their entropies exist, then for any real numbers $c_{1},c_{2},\cdots ,c_{n}$, we have*

(37) $$\begin{array}{c} H\left[c_{1}\xi_{1}+c_{2}\xi_{2}+\cdots +c_{n}\xi_{n}\right]=|c_{1}|H\left[\xi_{1}\right]+|c_{2}|H\left[\xi_{2}\right]+\cdots +\left|c_{n}\right|H\left[\xi_{n}\right]. \end{array}$$

**Proof** **of Theorem 4.** Without loss of generality, we only demonstrate the case n=2. Suppose that $c_{1}$ and $c_{2}$ are two real numbers, and $\xi_{1}$ and $\xi_{2}$ are two independent regular LR fuzzy intervals with ICDs, $\Phi_{1}^{-1}$ and $\Phi_{2}^{-1}$, respectively. If $c_{1}\geq 0$ and $c_{2}\geq 0$, it follows from Theorem 3 that

(38) $$\begin{array}{cc} H[c_{1}\xi_{1}+c_{2}\xi_{2}] & =\int_{0}^{1}\left(c_{1}\Phi_{1}^{-1}\left(\alpha \right)+c_{2}\Phi_{2}^{-1}\left(\alpha \right)\right)\ln\frac{\alpha }{1-\alpha }d\alpha \\ & =c_{1}\int_{0}^{1}\Phi_{1}^{-1}\left(\alpha \right)\ln\frac{\alpha }{1-\alpha }d\alpha +c_{2}\int_{0}^{1}\Phi_{2}^{-1}\left(\alpha \right)\ln\frac{\alpha }{1-\alpha }d\alpha \\ & =c_{1}H\left[\xi_{1}\right]+c_{2}H\left[\xi_{2}\right]=|c_{1}|H\left[\xi_{1}\right]+\left|c_{2}\right|H\left[\xi_{2}\right]. \end{array}$$

Similarly, if $c_{1}\leq 0$ and $c_{2}\geq 0$, we have

(39) $$\begin{array}{cc} H[c_{1}\xi_{1}+c_{2}\xi_{2}] & =\int_{0}^{1}\left(c_{1}\Phi_{1}^{-1}\left(1-\alpha \right)+c_{2}\Phi_{2}^{-1}\left(\alpha \right)\right)\ln\frac{\alpha }{1-\alpha }d\alpha \\ & =-c_{1}\int_{0}^{1}\Phi_{1}^{-1}\left(\alpha \right)\ln\frac{\alpha }{1-\alpha }d\alpha +c_{2}\int_{0}^{1}\Phi_{2}^{-1}\left(\alpha \right)\ln\frac{\alpha }{1-\alpha }d\alpha \\ & =-c_{1}H\left[\xi_{1}\right]+c_{2}H\left[\xi_{2}\right]=|c_{1}|H\left[\xi_{1}\right]+\left|c_{2}\right|H\left[\xi_{2}\right]. \end{array}$$

It is easy to derive that the equality $H\left[c_{1}\xi_{1}+c_{2}\xi_{2}\right]=|c_{1}|H\left[\xi_{1}\right]+\left|c_{2}\right|H\left[\xi_{2}\right]$ also holds for the other two cases (i.e., $c_{1}\;\geq$ 0 and $c_{2}\;\leq$ 0; $c_{1}\;\leq$ 0 and $c_{2}\;\leq$ 0). □

**Example** **14.** *Let $\xi_{1}∼T\left(2,3,1,1\right)$ and $\xi_{2}∼P\left(4,5,2,2\right)$. In view of Theorem 4 and Examples 9 and 10, the entropy of $6\xi_{1}+2\xi_{2}$ can be derived as*

(40) $$\begin{array}{c} H\left[6\xi_{1}+2\xi_{2}\right]=6H\left[\xi_{1}\right]+2H\left[\xi_{2}\right]=\frac{50}{3}+\frac{16\ln2}{3}-\frac{4\pi }{3}, \end{array}$$

*which is consistent with the result of Example 13.*

## 4. Simulation Algorithms for Entropy of Monotone Function

The calculation formula proposed in the above section can be used to directly calculate the entropy of linear function or simple nonlinear function. However, when the function becomes comparatively complicated or the quantity of fuzzy variables contained in the function increases, it becomes difficult to directly derive the entropy of function via the formula. In this case, designing effective simulation algorithms is a general and better choice instead of computing straightforwardly. This section first introduces two existing simulation algorithms by the stochastic discretization process, and then separately proposes two new simulation algorithms through the methods of uniform discretization and numerical integration, respectively.

### 4.1. FSE

By using the stochastic discretization simulation method introduced in [32], Huang [33] proposed the FSE through randomly generating the MF and CF in the light of the extension principle of Zadeh.

FSE in [33] is employed to approximate the fuzzy entropy $H[f\left(\xi_{1},\xi_{2},\cdots ,\xi_{n}\right)]$, where *f* is a real-valued function, and $\xi_{1},\xi_{2},\cdots ,\xi_{n}$ are independent fuzzy variables. Let $u_{1s},u_{2s},\cdots ,u_{ns}$ (s=1,2,⋯,K) be respectively generated from the ϵ-level sets of $\xi_{1},\xi_{2},\cdots ,\xi_{n}$, where *K* is a sufficiently large integer, and ϵ is a sufficiently small positive number. Denote $u_{s}=\left(u_{1s},u_{2s},\cdots ,u_{ns}\right)$ and $v_{s}=\mu_{1}\left(u_{1s}\right)\wedge \mu_{2}\left(u_{2s}\right)\wedge \cdots \wedge \mu_{n}\left(u_{ns}\right)$ for s=1,2,⋯,K. Thus, the credibility function can be approximately formulated as

(41) $$L=\frac{1}{2}\left(\max_{1\leq s\leq K}\left\{v_{s}|f\left(u_{s}\right)=r\right\}+1-\max_{1\leq s\leq K}\left\{v_{s}|f\left(u_{s}\right)\neq r\right\}\right),$$

which can be simplified as

(42) $$L=\frac{1}{2}\max_{1\leq s\leq K}\left\{v_{s}|f\left(u_{s}\right)=r\right\}.$$

Subsequently, by approximating the integration based on the sample points generated above, the detailed fuzzy simulation procedure of FSE is designed and described in Algorithm 1 below. Please note that in this simulation algorithm, there are two approximation processes which may produce errors, including randomly generating the discrete MF and randomly simulating the integration.

**Algorithm 1** Fuzzy Simulation for Entropy, FSE, Huang [33]
**Step 1.**
Initialize the numbers of sample points *K* and integration points *N*, and a sufficiently small positive number ϵ. Set H=0 and g=0.
**Step 2.**
Randomly generate real numbers $u_{is}$ such that $\mu_{i}\left(u_{is}\right)\geq \epsilon$, i=1,2,⋯,n,s=1,2,⋯,K, respectively.
**Step 3.**
Set $p=\min_{1\leq s\leq K}f\left(u_{s}\right)$, and $q=\max_{1\leq s\leq K}f\left(u_{s}\right)$.
**Step 4.**
Randomly generate *r* from [p,q].
**Step 5.**
Calculate $H_{g}=-t\ln t-\left(1-t\right)\ln\left(1-t\right)$, where $t=\mathrm{Cr}\{f\left(\xi_{1},\xi_{2},\cdots ,\xi_{n}\right)=r\}$ is estimated via Equation (42).
**Step 6.**
Set $H\leftarrow H+H_{g}$. If g<N, let g=g+1 and go to Step 4.
**Step 7.**
Return H·(q−p)/N as the estimated value of $H[f\left(\xi_{1},\xi_{2},\cdots ,\xi_{n}\right)]$.

Li [21] also developed an algorithm FSE* to simulate the entropy of function, $H[f\left(\xi_{1},\xi_{2},\cdots ,\xi_{n}\right)]$, in which the process of randomly generating sample points of continuous fuzzy numbers was the same as FSE. The essential difference between the two algorithms was the calculation of the joint credibility function $\mathrm{Cr}\{f\left(\xi_{1},\xi_{2},\cdots ,\xi_{n}\right)=x\}$. In FSE, the credibility function is estimated via Equation (42), whereas in FSE*, it is assumed to be predetermined by the decision-maker, which is impractical and difficult to perform.

### 4.2. UDA

Since FSE and FSE* both stemmed from the stochastic discretization process in [32], which has been verified by Miao et al. [34] to bring potential significant errors in most cases. To provide a superior approximate value of $H[f\left(\xi_{1},\xi_{2},\cdots ,\xi_{n}\right)]$, this section innovatively proposes a novel simulation method by performing the sampling experiments uniformly and then acquiring the fairly accurate membership degrees on account of the following theorem.

**Theorem** **5.** *(Miao et al. [34]) Let $\xi_{1},\xi_{2},\cdots ,\xi_{n}$ be independent regular LR fuzzy intervals. If the continuous function $f(x_{1},x_{2},\cdots ,x_{n})$ is strictly increasing in regard to $x_{1},x_{2},\cdots ,x_{h}$ and strictly decreasing in regard to $x_{h+1},x_{h+2},\cdots ,x_{n}$, then the MF of the fuzzy interval $f(\xi_{1},\xi_{2},\cdots ,\xi_{n})$ is*

(43) $$\begin{array}{c} \mu \left(x\right)=\mu_{1}\left(x_{1}\right){|}_{x=f\left(x_{1},x_{2},\cdots ,x_{n}\right),\;\left(x_{1},x_{2},\cdots ,x_{n}\right)\;\in \;L\;or\;R}, \end{array}$$

*where $\mu_{1}$ is the MF of $\xi_{1}$,*

(44) $$\begin{array}{c} L=\{\left(\xi_{1}^{L}\left(\alpha \right),\cdots ,\xi_{h}^{L}\left(\alpha \right),\xi_{h+1}^{R}\left(\alpha \right),\cdots ,\xi_{n}^{R}\left(\alpha \right)\right):0\leq \alpha \leq 1\},\\ R=\{\left(\xi_{1}^{R}\left(\alpha \right),\cdots ,\xi_{h}^{R}\left(\alpha \right),\xi_{h+1}^{L}\left(\alpha \right),\cdots ,\xi_{n}^{L}\left(\alpha \right)\right):0\leq \alpha \leq 1\}, \end{array}$$

*and $\left[\xi_{i}^{L}\left(\alpha \right),\xi_{i}^{R}\left(\alpha \right)\right]$ is the α-level of $\xi_{i}$, i=1,2,⋯,n.*

Theorem 5 tells us that the exact membership degree of any point in the two sets L or R can be achieved by means of that of the first fuzzy variable, $\mu_{1}\left(x_{1}\right)$, which motivates us to discretize the continuous fuzzy interval $f(\xi_{1},\xi_{2},\cdots ,\xi_{n})$ by generate sample points uniformly from L or R, instead of the stochastic discretization process used in FSE and FSE*.

Suppose that $f(x_{1},x_{2},\cdots ,x_{n})$ is a continuous function which is strictly increasing in regard to $x_{1},\cdots ,x_{h}$ and strictly decreasing in regard to $x_{h+1},\cdots ,x_{n}$, and $\xi_{i}∼{\left({\underline{a}}_{i},{\bar{a}}_{i},\beta_{i},\delta_{i}\right)}_{LR}$ are regular LR fuzzy intervals, i=1,2,⋯,n. Then separately divide the two close intervals $\left[{\underline{a}}_{i}-\beta_{i},{\underline{a}}_{i}\right]$ and $\left[{\bar{a}}_{i},{\bar{a}}_{i}+\delta_{i}\right]$ into *k* equal pieces, and denote the *s*-th point of $\left[{\underline{a}}_{i}-\beta_{i},{\underline{a}}_{i}\right]$ as $x_{is}^{L}$ and the (*k*-*s*)-th point of $\left[{\bar{a}}_{i},{\bar{a}}_{i}+\delta_{i}\right]$ as $x_{is}^{R}$, that is,

(45) $$\begin{array}{c} x_{is}^{L}={\underline{a}}_{i}-\beta_{i}+\beta_{i}\times \frac{s}{k},\;s=0,1,\cdots ,k,\\ x_{is}^{R}={\bar{a}}_{i}+\delta_{i}-\delta_{i}\times \frac{s}{k},\;s=0,1,\cdots ,k. \end{array}$$

For convenience, denote

(46) $$\begin{array}{c} X_{s}^{L}=\left(x_{1s}^{L},\cdots ,x_{hs}^{L},x_{h+1s}^{R},\cdots ,x_{ns}^{R}\right),\;s=0,1,\cdots ,k,\\ X_{s}^{R}=\left(x_{1s}^{R},\cdots ,x_{hs}^{R},x_{h+1s}^{L},\cdots ,x_{ns}^{L}\right),\;s=0,1,\cdots ,k,\\ X=\{f\left(X_{s}^{L}\right),f\left(X_{s}^{R}\right),s=0,1,\cdots ,k\}. \end{array}$$

Thus, based on Theorem 5, for any r∈X, the credibility function of $f(\xi_{1},\xi_{2},\cdots ,\xi_{n})$, $\mathrm{Cr}\{f(\xi_{1},\xi_{2},\cdots ,$$\xi_{n})=r\}$, can be well approximated by the following formula

(47) $$C=\left\{\begin{array}{cc} \frac{1}{2}\mu_{1}\left(x_{1s}^{L}\right), & \mathrm{if}\;r<f\left(X_{k}^{L}\right)\;\mathrm{and}\;r=f\left(X_{s}^{L}\right)\\ 0.5, & \mathrm{if}\;r\in [f\left(X_{k}^{L}\right),f\left(X_{k}^{R}\right)]\\ \frac{1}{2}\mu_{1}\left(x_{1s}^{R}\right), & \mathrm{if}\;r>f\left(X_{k}^{R}\right)\;\mathrm{and}\;r=f\left(X_{s}^{R}\right). \end{array}\right.$$

**Figure 9:**
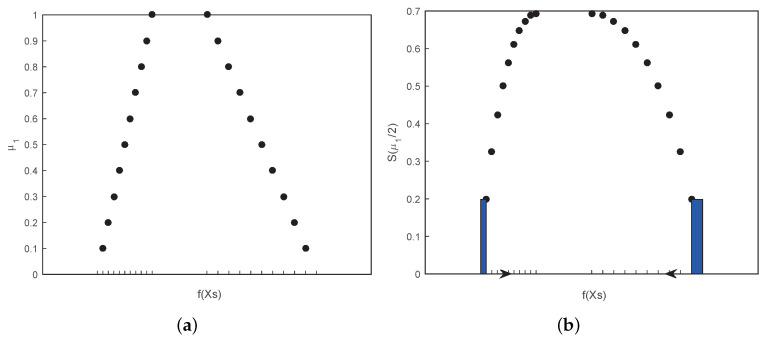
(**a**) Discretization process of UDA. The horizontal axis represents the function of fuzzy variables, and the left and right sides of this set variable are respectively cut into uniform parts. The vertical axis represents the membership of the first fuzzy variable. (**b**) Calculation process of UDA. The small area with color is the simulation value, the bottom is the spacing of the adjacent set function and the height is the integrand function, adding from the two sides to the middle.

Subsequently, according to Equations (33) and (47), through the discretization method mentioned above, the UDA is designed in Algorithm 2 as follows. Figure 9a,b explain the simulation process of UDA with the trapezoidal fuzzy numbers when *k* is set to 10.

**Algorithm 2** Uniform Discretization Algorithm, UDA
**Step 1.**
Initialize the number of sample points *K*. Set H=0, s=0, and $k=\frac{K}{2}$.
**Step 2.**
Set s=s+1 and calculate $H=H+\left(-t\ln t-\left(1-t\right)\ln\left(1-t\right)\right)w_{s}$, where $t=\frac{1}{2}\mu_{1}\left(x_{1s}^{L}\right)$, $w_{s}=f\left(X_{s}^{L}\right)-f\left(X_{s-1}^{L}\right)$.
**Step 3.**
If s≤k, go to Step 2. Otherwise, set s=0 and go to Step 4.
**Step 4.**
Set s=s+1 and calculate $H=H+\left(-t\ln t-\left(1-t\right)\ln\left(1-t\right)\right)w_{k-s}$, where $t=\frac{1}{2}\mu_{1}\left(x_{1s}^{R}\right)$, $w_{k-s}=f\left(X_{s}^{R}\right)-f\left(X_{s-1}^{R}\right)$.
**Step 5.**
If s≤k, go to Step 4. Otherwise, go to Step 6.
**Step 6.**
Calculate $H=H+S\left(0.5\right)w_{k}$, where $w_{k}=f\left(X_{k}^{R}\right)-f\left(X_{k}^{L}\right)$.
**Step 7.**
Return *H* as the estimated value of $H[f\left(\xi_{1},\xi_{2},\cdots ,\xi_{n}\right)]$.

Although both FSE and UDA estimate the entropy of function by using the discretization process and the calculation formula in Equation (33), there are two underlying differences between them. One is the sampling method for discretization of continuous fuzzy intervals. FSE generates sample points randomly with a less accurate MF, while UDA generates sample points uniformly, outputting a pretty accurate MF. The other is the integration simulation process, the performance of which will be demonstrated in the next section.

### 4.3. NIA

As proved above, the entropy of function of regular LR fuzzy intervals can be calculated via an integration involving its ICD in integrand (see Theorem 3). Based on this calculation formula (i.e., Equation (27)), a NIA is suggested to simulate the entropy of monotone function efficiently.

**Figure 10:**
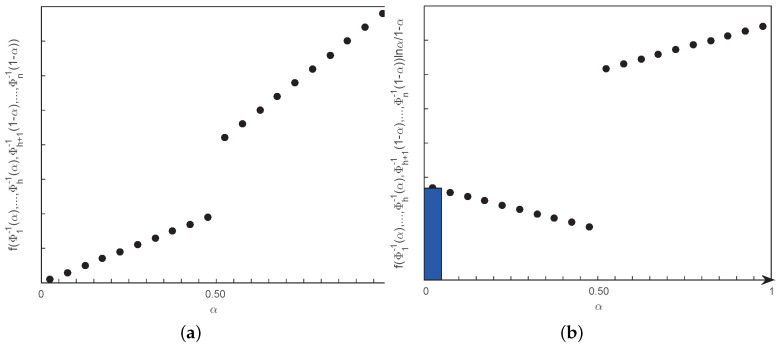
(**a**) Discretization process of NIA. The abscissa of the discrete points is the midpoint of the adjacent sample points, and the ordinate is the value of $f\left(\Phi_{1}^{-1}\left(\alpha \right),\cdots ,\Phi_{h}^{-1}\left(\alpha \right),\Phi_{h+1}^{-1}\left(1-\alpha \right),\cdots ,\Phi_{n}^{-1}\left(1-\alpha \right)\right)$ corresponding to the midpoint. (**b**) Calculation process of NIA. The small area with color is the simulation value, the bottom is the spacing of the adjacent sample points and the height is the integrand function, adding from 0 to 1.

Suppose that $f(x_{1},x_{2},\cdots ,x_{n})$ is strictly increasing in regard to $x_{1},\cdots ,x_{h}$ and strictly decreasing in regard to $x_{h+1},\cdots ,x_{n}$, and $\xi_{1},\xi_{2},\cdots ,\xi_{n}$ are regular LR fuzzy intervals with ICDs, $\Phi_{1}^{-1},\Phi_{2}^{-1},\cdots ,\Phi_{n}^{-1}$, respectively. Then in view of Theorem 3, the entropy can be simulated as the numerical integration of function $f\left(\Phi_{1}^{-1}\left(\alpha \right),\cdots ,\Phi_{h}^{-1}\left(\alpha \right),\Phi_{h+1}^{-1}\left(1-\alpha \right),\cdots ,\Phi_{n}^{-1}\left(1-\alpha \right)\right)\ln\frac{\alpha }{1-\alpha }$. To further reduce the deviation, the median values of two sample points are used to simulate the above integrand. More concretely, divide the close interval [0,1] into *K* equal pieces, and integrate the above function for α=(2s−1)/2K, s=1,2,⋯,K. Taking the trapezoidal fuzzy numbers as an example, Figure 10a,b explain the simulation process of NIA when *K* is set to 20. The detailed procedure is described in Algorithm 3 as follows.

**Algorithm 3** Numerical Integration Algorithm, NIA
**Step 1.**
Initialize the number of sample points *K*. Set H=0 and s=1.
**Step 2.**
Set α=(2s−1)/2K. For each 1≤i≤h, let $x_{i}=\Phi_{i}^{-1}\left(\alpha \right)$, and for each h+1≤i≤n, let $x_{i}=\Phi_{i}^{-1}\left(1-\alpha \right)$.
**Step 3.**
Reset $H=H+f\left(x_{1},x_{2},\cdots ,x_{n}\right)\ln\frac{\alpha }{1-\alpha }/K$ and s=s+1.
**Step 4.**
If s≤K, go to Step 2. Otherwise, return *H* as the estimated value of $H[f\left(\xi_{1},\xi_{2},\cdots ,\xi_{n}\right)]$.

NIA provides a new method to simulate the entropy of monotone function, which is slightly different from FSE and UDA as it substantially simulates the entropy through the ICD rather than the credibility function. As seen from the process of the algorithms, compared to UDA, the accuracy of the results of NIA is apparently improved since the simulation errors only come from the process of the integral simulation. When *K* tends to be infinite, the simulation results of NIA will definitely approach the real value of the entropy $H[f\left(\xi_{1},\xi_{2},\cdots ,\xi_{n}\right)]$.

## 5. Numerical Experiments

In this section, a series of experiments on different types of fuzzy intervals with same parameters $\underline{a},\bar{a},\beta ,\delta$ shown in Table 3 are conducted in *C* language and run in the same environment, a windows 10 platform PC with processor speed of 2.50 GHz.

Next, we will give four examples to compare the efficiency of FSE, UDA, and NIA from three perspectives, separately studying the influence of the changes in the number of fuzzy intervals, the types of fuzzy intervals and functions on the algorithm results, and further explore the applicable conditions of the three algorithms. Besides, the parameters involved in the algorithms are fixed, in which the numbers of sample points and integration points are both set to be 10,000. To reasonably compare the results simulated by FSE, UDA, and NIA, the simulation error is measured by error=|simulationvalue−exactvalue|/exactvalue×100%, where the exact results in all examples are calculated according to Theorem 3.

**Example** **15.** *This example is used to verify the stability and precision of algorithms when calculating the entropy of a single fuzzy variable, H[ξ].*

Assume that $\xi_{1}$ is an LR fuzzy interval with $\underline{a},\bar{a},\beta ,\delta$ listed in Table 3. Firstly, we run FSE, UDA, and NIA 20 times on the trapezoidal fuzzy number to figure out the estimated entropy $H[\xi_{1}]$, and record their simulation results in Table 4, respectively.

The mean, variance, and coefficient of variation are obtained by descriptive statistics. Here, the coefficient of variation is the ratio of standard deviation to mean, reflecting the dispersion degree of observations. From Table 4, we can get the conclusion that the algorithms of UDA and NIA are extremely stable and constant, since the two algorithms will output a unique result according to their respective principles, and the stability of FSE is acceptable as well with a quite small coefficient of variation 0.002387.

**Figure 11:**
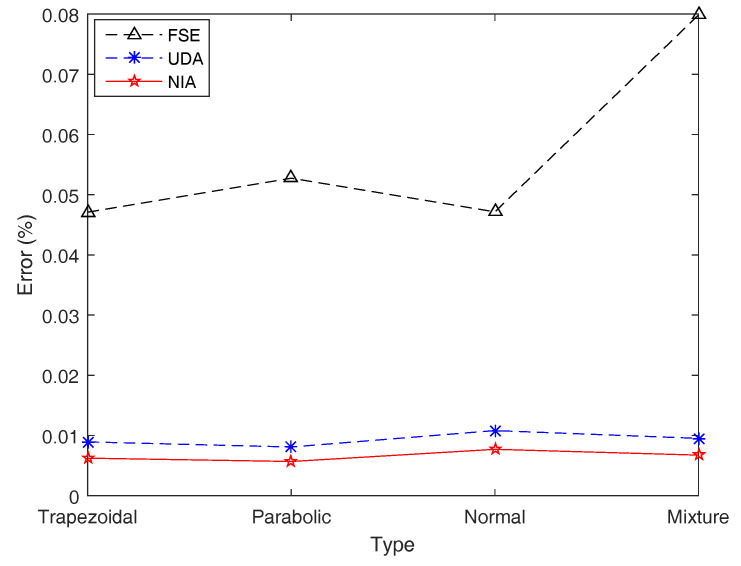
Simulation errors of FSE, UDA, and NIA in Example 15. Simulation errors of UDA and NIA are close, and those of FSE are acceptable.

Subsequently, by employing the three simulation algorithms, the simulation and exact results for entropy of $\xi_{1}$ on four types of LR fuzzy intervals are obtained and shown in Table 5 and Figure 11, where each simulation result is reported as the average of 20 simulated trials.

Table 5 and Figure 11 show that NIA performs best on simulation results compared with the other two algorithms, while UDA runs fastest for its algorithm principle using the first membership value to represent all, which can be verified by the following examples. For an individual fuzzy interval, although the simulation errors of FSE are several times more than those of the other two algorithms, the precision of FSE is still acceptable with a maximum error 0.079867%. In terms of the speed of operation, the maximum computing time of FSE is 0.6928s, which is far worse than UDA and NIA.

**Example** **16.** *This example is used to verify the stability and precision of algorithms when calculating the entropy of function, $H[f\left(\xi_{1},\xi_{2},\cdots ,\xi_{n}\right]$, where $\xi_{i}$ are LR fuzzy intervals belonging to the same type, i=1,2,⋯,n.*

Let $f\left(x_{1},x_{2},\cdots ,x_{10}\right)=x_{1}+x_{2}+\cdots +x_{10}$, and $\xi_{1},\xi_{2},\cdots ,\xi_{10}$ be regular LR fuzzy intervals with $\underline{a},\bar{a},\beta ,\delta$ listed in Table 3. Firstly, analogously, we run FSE, UDA, and NIA 20 times on the trapezoidal fuzzy numbers to estimate the entropy $H\left[f\left(\xi_{1},\xi_{2},\cdots ,\xi_{n}\right)\right]=H\left[\xi_{1}+\xi_{2}+\cdots +\xi_{10}\right]$, and then record their simulation results in Table 6, together with some descriptive statistics.

From Table 6, the stability of UDA and NIA keeps perfect, exporting only one result for all trials, but that of FSE becomes weaker obviously when compared to Example 15. To avoid contingency and enhance persuasiveness, in the following experiments, we always run FSE 20 times and then report their average as the final result.

**Figure 12:**
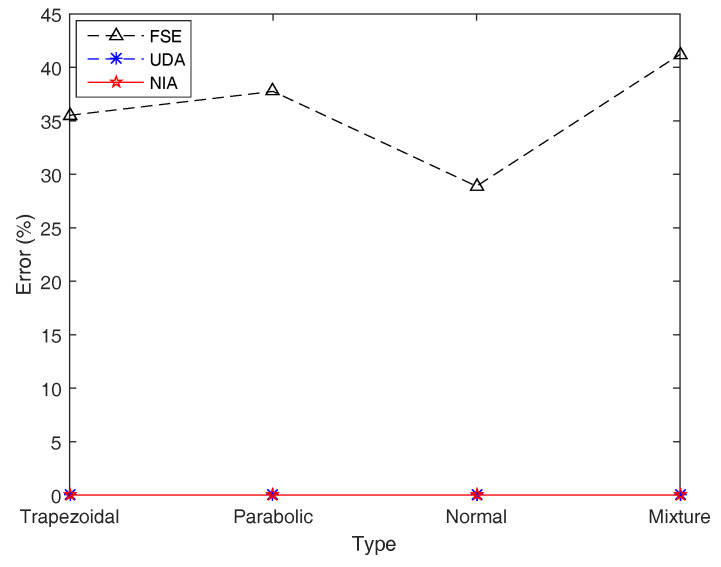
Simulation errors of FSE, UDA, and NIA in Example 16. Simulation errors of UDA and NIA are close, and those of FSE are poor.

Furthermore, for exploring the accuracy of the three simulation algorithms, the (average) simulation results of the entropy $H[\xi_{1}+\xi_{2}+\cdots +\xi_{10}]$ on four types of LR fuzzy intervals by running FSE, UDA, and NIA are presented in Table 7 and Figure 12.

By observing Table 7 and Figure 12, with regard to the addition function *f* with respect to the same type of LR fuzzy intervals, NIA still performs best at simulation results, followed by UDA, and FSE has the worst performance, with a maximum error of 41.208792%, while the highest errors of UDA and NIA are just 0.009314% and 0.007314%, respectively. In addition, the comparative analysis of the running time demonstrates that UDA is still the most advantageous, followed by NIA, while FSE is far behind, hundreds of times slower.

**Example** **17.** *This example is used to verify the precision of algorithms when calculating the entropy of function, $H[f\left(\xi_{1},\xi_{2},\cdots ,\xi_{n}\right]$, where $\xi_{i}$ are LR fuzzy intervals belonging to different types, i=1,2,⋯,n.*

**Figure 13:**
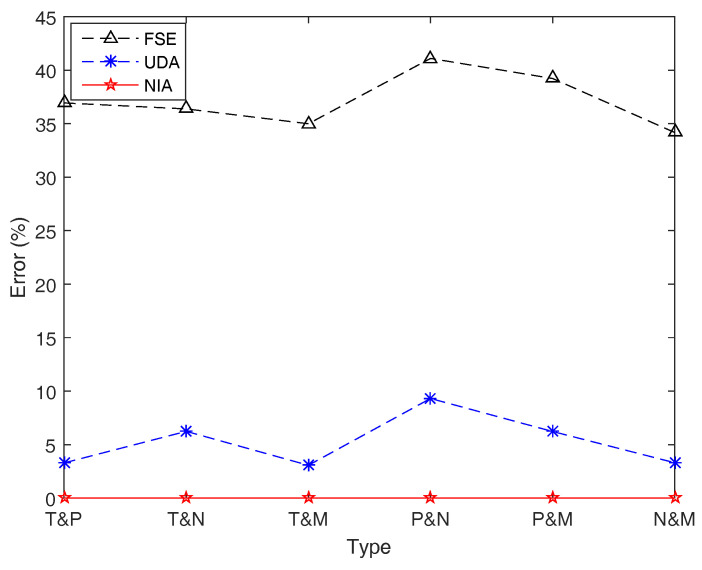
Simulation errors of FSE, UDA, and NIA in Example 17. Simulation errors of UDA are worse than those of NIA, and those of FSE are quite large up to 41%.

Let $f\left(x_{1},x_{2},\cdots ,x_{10}\right)=x_{1}+x_{2}+\cdots +x_{10}$, and $\xi_{1},\xi_{2},\cdots ,\xi_{10}$ be regular LR fuzzy intervals with $\underline{a},\bar{a},\beta ,\delta$ listed in Table 3. To investigate the performance of the three algorithms for calculating the entropy of function involving different types of fuzzy intervals, in this example, we assume that the first five fuzzy intervals, $\xi_{1},\xi_{2},\cdots ,\xi_{5}$, belong to the same type, and the last five, $\xi_{6},\xi_{7},\cdots ,\xi_{10}$, are another kind of the same type. For instance, $\xi_{i},i=1,2,\cdots ,5$, are all trapezoidal fuzzy numbers, and $\xi_{i},i=6,7,\cdots ,10$, are all parabolic fuzzy intervals, which is denoted as “T& P” for simplicity. As for different combinations of different types of LR fuzzy intervals, FSE, UDA, and NIA are run to export the (average) simulation results of the entropy $H[\xi_{1}+\xi_{2}+\cdots +\xi_{10}]$, and the results are shown in Table 8 and Figure 13.

From and Table 8 and Figure 13, we can find that NIA has an overwhelming superiority on accuracy compared with the other two algorithms in this case, while FSE still performs worst with the maximal error 41.098743%. It is worth noting that the accuracy of UDA is reduced when the function *f* includes different types of fuzzy intervals. In this situation, the method which helps UDA take an absolute advantage in speed, that is, to use the membership of the first fuzzy interval to represent all, may lead to a big error up to 9.312994%. In other words, UDA cannot effectively estimate the entropy of function involving different combinations of different types of fuzzy intervals.

**Example** **18.** *This example is used to further verify the precision of algorithms when calculating the entropy of function, $H[f\left(\xi_{1},\xi_{2},\cdots ,\xi_{n}\right]$, where $\xi_{i}$ belong to different types, i=1,2,⋯,n, and f is a more complex function compared with Example 17.*

**Figure 14:**
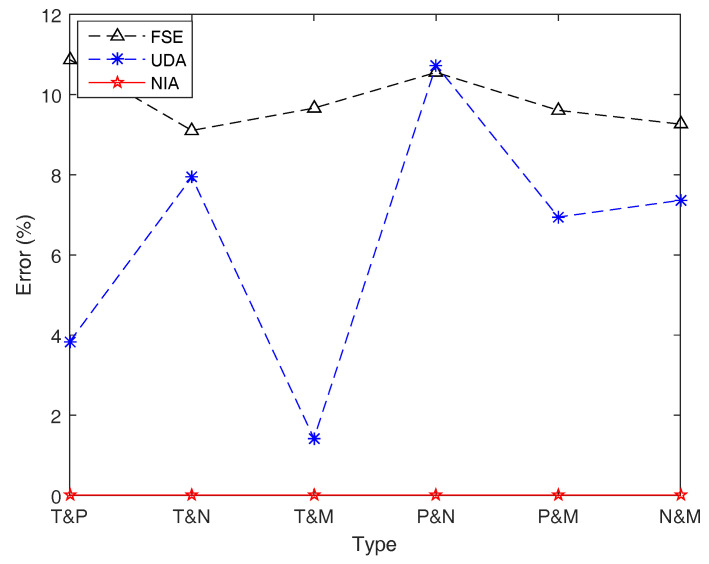
Simulation errors of FSE, UDA, and NIA in Example 18. Simulation errors of FSE and UDA are obviously greater than those of NIA.

Let $f\left(x_{1},x_{2},\cdots ,x_{10}\right)=x_{1}\wedge x_{2}\wedge \cdots \wedge x_{10}$, and the other conditions are the same as Example 17. The (average) simulation results for entropy $H[\xi_{1}\wedge \xi_{2}\wedge \cdots \wedge \xi_{10}]$ with respect to different combinations of different types of LR fuzzy intervals are shown in Table 9 and Figure 14.

From Table 9 and Figure 14, we can get similar conclusions as the previous example. In terms of accuracy, NIA performs best, while FSE and UDA perform not very well. For NIA, both accuracy and the operation speed are extremely excellent. However, for UDA, although it has an outstanding running speed, it cannot figure out an acceptable estimation result with good precision when different types of fuzzy variables are involved in the function. As for FSE, there is no reason to choose it either in terms of accuracy or speed.

From Examples 15–18, it can be concluded that NIA is the best algorithm when considering the precision and stability, whether it is simulating the entropy of individual fuzzy interval or monotone function. All the estimation errors of the results by running NIA are less than 0.008%, and the maximal running time is less than 0.007s. The speed of UDA is most advantageous, nearly ten times faster than NIA, but its simulation results are rather poor when the estimated function involves different types of fuzzy intervals, and it is only applicable to the simulation of functions regarding the same type of fuzzy intervals. Compared with the proposed two new algorithms for entropy simulation, the existing algorithm FSE is bad in any case, which will report the estimation result with the maximal error of greater than 40%.

## 6. Conclusions

Motivated by the limitation of the current literature, this paper aims to improve the calculation of entropy of function, $H[f\left(\xi_{1},\xi_{2},\cdots ,\xi_{n}\right)]$, to extend the application scope of fuzzy entropy in the decision-making optimization problems. In our paper, all the fuzzy variables $\xi_{i}$ are assumed to be regular LR fuzzy intervals, a type of commonly used fuzzy variables, involving triangular fuzzy numbers and trapezoidal fuzzy numbers as special cases. In addition, the function *f* is considered to be a strictly monotone function with respect to all the fuzzy variables, and most of the functions involved in real application problems meet this condition. Thus, the simplified calculation formulas as well as the simulation algorithms based on the formulas can provide a valuable reference to entropy optimization from a new perspective, supplementing the existing measures such as mean and variance to extend the applications of LR fuzzy intervals.

Table 10 summarizes most work referred to in this paper, in which the colored methods (i.e., the calculation formula Equation (34), the simulation algorithms UDA and NIA) are contributions of our paper, while Equation (33) and FSE come from literature [19,33], respectively. The relevant research can be mostly divided into two parts from the perspective of processing objects. On the one hand, as for the entropy of linear or simple nonlinear function *f*, the calculation formulas Equations (33) and (34) can be directly used to obtain the entropy $H[f\left(\xi_{1},\xi_{2},\cdots ,\xi_{n}\right)]$. Compared with Equation (33), our calculation formula Equation (34) replaced the credibility function by the ICD, which greatly simplifies the integrand by removing the function *S* contained in the original definition (see Definition 8). Correspondingly, Equation (34) can solve all the linear functions and some simple nonlinear functions effortlessly, but Equation (33) can only deal with linear functions $f\left(\xi_{1},\xi_{2},\cdots ,\xi_{n}\right)=a_{1}\xi_{1}+a_{2}\xi_{2}+\cdots +a_{n}\xi_{n}$, with the constraint that $\xi_{i}$ belong to the same type of fuzzy variables, e.g., they are all triangular or trapezoidal fuzzy numbers.

On the other hand, regarding complicated nonlinear function *f*, using Equation (34) becomes incredibly difficult even with the help of software packages such as MATLAB. Therefore, fuzzy simulation, a widely used technique for obtaining an approximation, is introduced to deal with this issue based on the above calculation formulas and some discretization sampling procedures. The representative simulation algorithm in the current literature, FSE, was proposed by Huang [33], which is based on Equation (33) and the stochastic discretization process presented by Liu [32]. Due to the complexity of Equation (33) and the inaccuracy of Liu [32]’s method, FSE can return satisfactory results stably only when calculating the entropy of an individual fuzzy variable, H[ξ], with an estimation error of less than 0.08% (see Table 9 in Example 15), whereas for other situations, the performance of FSE is rather poor, possibly reporting results with errors of greater than 40% (see Table 7, Table 8 and Table 9 in Examples 16–18), and simultaneously the running time is hundreds of times more than our algorithms.

The proposed two algorithms in this paper have different mechanisms. Inspired by Theorem 5 presented by Miao et al. [34], the first proposed simulation algorithm, UDA, generates sample points from the possible range of each fuzzy variable uniformly, and then estimates the entropy of function via Equation (33) by using the membership of the first fuzzy variable, $\mu_{\xi_{1}}\left(x_{1}\right)$, to approximate that of $f(\xi_{1},\xi_{2},\cdots ,\xi_{n})$. For this reason, UDA has the absolute advantage in computing speed. The running time of UDA in all the examples is less than 0.0004 s, ten times faster than NIA and hundreds of times than FSE. However, this simplification may also cause potential errors of greater than 10% when the MFs of $\xi_{i}$ differ greatly from each other (see Table 8 and Table 9 in Examples 17–18).

The second simulation algorithm designed in this paper, NIA, is based on Equation (34) represented by the ICD. After dividing the close interval [0,1] into sufficiently small equal pieces, NIA computes the numerical integration of each area via the median values of two sample points. Since the error of NIA just comes from the integration simulation, the performance of NIA is extremely excellent both in accuracy and speed. Even though its operation speed is worse than UDA, the running time is also acceptable. Thus, as concerning the universality of applications, NIA will be recommended preferentially because of its highest accuracy (all errors are less than 0.008%), best stability (always output only one result), and acceptable speed (all computing times are less than 0.007s).

## Figures and Tables

**Table 1:** A summary of literature on calculation of fuzzy entropy of function, $H[f\left(\xi_{1},\cdots ,\xi_{n}\right)]$. Our paper improves the existing work and reduces the computation complexity via an explicit expression for both linear and nonlinear functions involving different types of fuzzy variables.

Literature	Types of variables $\xi_{i}$		Type of function *f*		Expression of $H[f\left(\xi_{1},\cdots ,\xi_{n}\right)]$	
	Same	Different	Linear	Nonlinear	Explicit	Inexplicit
Huang [24]	*√*		*√*		*√*	
Li et al. [25]	*√*		*√*		*√*	
Jalota et al. [26]	*√*		*√*		*√*	
Zhou et al. [27]	*√*		*√*		*√*	
Deng et al. [28]	*√*		*√*		*√*	
Yari et al. [29]	*√*		*√*		*√*	
Li [21]	*√*		*√*		*√*	
Li [21]	*√*	*√*	*√*			*√*
Our Paper	*√*	*√*	*√*	*√*	*√*	

**Table 2:** A summary of current simulation algorithms for entropy of function $H[f\left(\xi_{1},\cdots ,\xi_{n}\right)]$. Our paper improves the existing work with extremely high accuracy and short computing time by presenting the uniformly discretization algorithm and the numerical integration algorithm for monotone functions.

Literature	Algorithm	Function *f*		Principle	Accuracy	
		General	Monotone		High	Low
Huang [33]	FSE	*√*		Stochastic discre-tization process		*√*
Li [21]	FSE*	*√*		Stochastic discre-tization process		*√*
Our Paper	UDA		*√*	Uniform discre-tization process	*√* (same type)	*√* (others)
Our Paper	NIA		*√*	Numerical integration	*√*	

**Table 3:** Parameter settings of LR fuzzy intervals $\xi_{1},\xi_{2},\cdots ,\xi_{10}$ used in Examples 15–18. Please note that $\xi_{1},\xi_{2},\cdots ,\xi_{10}$ in each example represents different types of LR fuzzy intervals, but with the same parameters, $\underline{a},\bar{a},\beta ,\delta$, for the sake of simplicity on the symbols.

No.	$\underline{a},\;\bar{a},\;\beta ,\;\delta$	No.	$\underline{a},\;\bar{a},\;\beta ,\;\delta$
$\xi_{1}$	3, 5, 2, 3	$\xi_{6}$	4, 8, 1, 2
$\xi_{2}$	4, 7, 2, 2	$\xi_{7}$	3, 5, 1, 1
$\xi_{3}$	5, 6, 3, 4	$\xi_{8}$	5, 7, 1, 2
$\xi_{4}$	7, 8, 4, 1	$\xi_{9}$	2, 6, 1, 2
$\xi_{5}$	3, 4, 2, 3	$\xi_{10}$	3, 6, 1, 4

**Table 4:** Results and their descriptive statistics by running FSE, UDA, and NIA 20 times in Example 15. The results of UDA and NIA keep unchanged, while those of FSE fluctuate with a small coefficient of variation.

Algorithm	FSE		UDA		NIA	
20 results of $H[\xi_{1}]$	3.881256	3.883001	3.886641	3.886641	3.886052	3.886052
	3.897752	3.890260	3.886641	3.886641	3.886052	3.886052
	3.899839	3.861699	3.886641	3.886641	3.886052	3.886052
	3.889567	3.880707	3.886641	3.886641	3.886052	3.886052
	3.880670	3.881301	3.886641	3.886641	3.886052	3.886052
	3.888158	3.885380	3.886641	3.886641	3.886052	3.886052
	3.892246	3.897574	3.886641	3.886641	3.886052	3.886052
	3.887110	3.868030	3.886641	3.886641	3.886052	3.886052
	3.881969	3.889256	3.886641	3.886641	3.886052	3.886052
	3.876045	3.877455	3.886641	3.886641	3.886052	3.886052
Mean	3.884464		3.886641		3.886052	
Variance	0.000086		0		0	
Coefficient of variation	0.002387		0		0	

**Table 5:** Comparative results in Example 15. FSE, UDA, and NIA are respectively run to simulate the entropy of a single fuzzy interval, $H[\xi_{1}]$, and their simulation results, running time and simulation errors are reported.

Variable Type of $\xi_{1}$		Trapezoidal	Parabolic	Normal	Mixture
Exact value		3.886294	4.279722	3.209027	3.637305
	Result	3.884464	4.281979	3.210540	3.640210
FSE	Time (s)	0.674900	0.681300	0.692800	0.687000
	Error (%)	0.047095	0.052737	0.047148	0.079867
	Result	3.886641	4.280068	3.209374	3.637651
UDA	Time (s)	0.000040	0.000340	0.000240	0.000230
	Error (%)	0.008929	0.008085	0.010813	0.009513
	Result	3.886052	4.279479	3.208780	3.637060
NIA	Time (s)	0.003700	0.004600	0.003400	0.004100
	Error (%)	0.006227	0.005678	0.007697	0.006736

**Table 6:** Results and their descriptive statistics by running FSE, UDA, and NIA 20 times in Example 16. The results of UDA and NIA keep unchanged, while those of FSE fluctuate with a small coefficient of variation.

Algorithm	FSE		UDA		NIA	
20 results of $H[\xi_{1}+\cdots +\xi_{10}]$	23.307949	24.033430	36.945296	36.945296	36.940132	36.940132
	23.942192	22.959617	36.945296	36.945296	36.940132	36.940132
	24.237903	25.027487	36.945296	36.945296	36.940132	36.940132
	23.309498	22.736498	36.945296	36.945296	36.940132	36.940132
	26.859123	25.222795	36.945296	36.945296	36.940132	36.940132
	23.594754	22.614552	36.945296	36.945296	36.940132	36.940132
	23.345227	24.837568	36.945296	36.945296	36.940132	36.940132
	24.326482	24.137683	36.945296	36.945296	36.940132	36.940132
	23.162334	22.738563	36.945296	36.945296	36.940132	36.940132
	21.964390	24.077345	36.945296	36.945296	36.940132	36.940132
Mean	23.821770		36.945296		36.940132	
Variance	1.178396		0		0	
Coefficient of variation	0.045569		0		0	

**Table 7:** Comparative results in Example 16. FSE, UDA, and NIA are respectively run to simulate the entropy of function, $H[\xi_{1}+\xi_{2}+\cdots +\xi_{10}]$, where $\xi_{i}$ are with the same type, and their simulation results, running time and simulation errors are reported.

Variable Type of $\xi_{i}$		Trapezoidal	Parabolic	Normal	Mixture
Exact value		36.942385	40.247176	31.253340	35.107841
	Result	23.821770	25.051830	22.224826	20.640324
FSE	Time (s)	0.5103000	0.5352000	0.5416000	0.5341000
	Error (%)	35.516428	37.755061	28.888157	41.208792
	Result	36.945296	40.250086	31.256251	35.110752
UDA	Time (s)	0.0002200	0.0002500	0.0002500	0.0002300
	Error (%)	0.0078800	0.0072300	0.0093140	0.0082920
	Result	36.940132	40.244923	31.251054	35.105569
NIA	Time (s)	0.0051000	0.0059000	0.0062000	0.0060000
	Error (%)	0.0060990	0.0055980	0.0073140	0.0064710

**Table 8:** Comparative results in Example 17. FSE, UDA, and NIA are respectively run to simulate the entropy of function, $H[\xi_{1}+\xi_{2}+\cdots +\xi_{10}]$, where $\xi_{i}$ are with different types, and their simulation results, running time, and simulation errors are reported.

Variable Type of $\xi_{i}$		T&P	T&N	T&M	P&N	P&M	N&M
Exact value		38.201353	34.775130	35.845825	36.820953	37.891648	32.324035
	Result	24.091137	22.123835	23.302385	21.688004	23.030277	21.278486
FSE	Time (s)	0.5094000	0.5084000	0.5042000	0.5116000	0.5101000	0.5286000
	Error (%)	36.936430	36.380295	34.992750	41.098743	39.220704	34.171319
	Result	36.945296	36.945296	36.945296	40.250086	40.250086	31.256251
UDA	Time (s)	0.0002200	0.0002200	0.0002200	0.0002500	0.0002500	0.0002500
	Error (%)	3.2879910	6.2405690	3.0672220	9.3129940	6.2241630	3.3033750
	Result	38.199100	34.772864	35.843563	36.818687	37.889386	32.321752
NIA	Time (s)	0.0054000	0.005800	0.0054000	0.0055000	0.0059000	0.0060000
	Error (%)	0.0058980	0.0065160	0.0063090	0.0061530	0.0059680	0.0070620

**Table 9:** Comparative results in Example 18. FSE, UDA, and NIA are respectively run to simulate the entropy of function, $H[\xi_{1}\wedge \xi_{2}\wedge \cdots \wedge \xi_{10}]$, where $\xi_{i}$ are with different types, and their simulation results, running time, and simulation errors are reported.

Variable Type of $\xi_{i}$		T&P	T&N	T&M	P&N	P&M	N&M
Exact value		3.158177	2.813635	2.994500	2.917810	3.021154	2.904143
	Result	2.814814	2.557482	2.705171	2.609910	2.730920	2.635169
FSE	Time (s)	0.546500	0.557900	0.547100	0.546200	0.552400	0.548000
	Error (%)	10.872190	9.103988	9.662014	10.552435	9.606726	9.261734
	Result	3.037040	3.037040	3.037040	3.230935	3.230935	2.690357
UDA	Time (s)	0.000260	0.000240	0.000240	0.000250	0.000250	0.000250
	Error (%)	3.835662	7.940085	1.420604	10.731508	6.943737	7.361414
	Result	3.158004	2.813461	2.994326	2.917636	3.020980	2.903969
NIA	Time (s)	0.005400	0.005400	0.005300	0.006100	0.006300	0.006200
	Error (%)	0.005478	0.006184	0.005811	0.005963	0.005759	0.005991

**Table 10:** A summary of the methods for calculating the entropy $H[f\left(\xi_{1},\xi_{2},\cdots ,\xi_{n}\right]$ referred to in this paper, including the calculation formulas and the simulation algorithms. The colored methods are contributions of the current paper.

Object	Means	Methodology	Mechanism	Characteristic
Linear or simple nonlinear function	Calculation formula	Equation (33)	credibility function	Complex operation (limiting to linear applications)
		**Equation (34)**	inverse credibility distribution	Simplified operation (extending to nonlinear applications)
Complicated nonlinear function	Simulation algorithm	FSE	Equation (33) & stochastic discretization	Low precision & speed (acceptable precision only for a single fuzzy variable)
		**UDA**	Equation (33) & uniform discretization	High speed (high precision only for the same type)
		**NIA**	Equation (34) & numerical integration	High precision & speed (high precision for all conditions)

## Abbreviations

The following abbreviations are used in this manuscript:

FSE	Fuzzy simulation for entropy
UDA	Uniform discretization algorithm
NIA	Numerical integration algorithm
MF	membership function
CD	credibility distribution
ICD	inverse credibility distribution
